# Supplementary material for: The Combination of a Human Biomimetic Liver Microphysiology System with BIOLOGXsym, a Quantitative Systems Toxicology (QST) Modeling Platform for Macromolecules, Provides Mechanistic Understanding of Tocilizumab- and GGF2-Induced Liver Injury
Source: Int J Mol Sci. 2023 Jun 2;24(11):9692. doi: 10.3390/ijms24119692 (PMC10253699; doi:10.3390/ijms24119692)
Supplement: Supplementary file 1 [file ijms-24-09692-s001.zip › ijms-2398822-supplementary.pdf]

Supplementary Information

The Combination of a Human Biomimetic Liver Microphysiology System with BIOLOGXsym, a Quantitative Systems Toxicology (QST) Modeling Platform for Macromolecules, Provides Mechanistic Understanding of Tocilizumab- and GGF2-Induced Liver Injury

James J. Beaudoin [1,†], Lara Clemens [1,†], Mark T. Miedel [2], Albert Gough [2], Fatima Zaidi [3], Priya Ramamoorthy [3], Kari E. Wong [3], Rangaprasad Sarangarajan [3], Christina Battista [1], Lisl K. M. Shoda [1], Scott Q. Siler [1], D. Lansing Taylor [2], Brett A. Howell [1], Lawrence A. Vernetti [2,*] and Kyunghee Yang [1,*]

[1] DILIsym Services Division, Simulations Plus Inc., Research Triangle Park, Durham, NC 27709, USA; scott.siler@simulations-plus.com (S.Q.S.)
[2] Department of Computational and Systems Biology, Drug Discovery Institute, University of Pittsburgh, Pittsburgh, PA 15219, USA; gough@pitt.edu (A.G.); dltaylor@pitt.edu (D.L.T.)
[3] Metabolon Inc., Durham, NC 27713, USA; sramamoorthy@metabolon.com (P.R.); kwong@metabolon.com (K.E.W.); rsarangarajan@metabolon.com (R.S.)
* Correspondence: vernetti@pitt.edu (L.A.V.); kyunghee.yang@simulations-plus.com (K.Y.); Tel.: +1-412-624-5425 (L.A.V.); Tel.: +1-984-444-8305 (K.Y.)
† These authors contributed equally to this work.

1. MATERIALS AND METHODS

1.1. LAMPS assays

1.1.1. The Liver Acinus Microphysiology System (LAMPS) model

Figure S1. The LAMPS is an experimental platform to model liver diseases and assess drug efficacy and safety. Schematic diagrams of the LAMPS showing the 4 liver cell types (hepatocytes, Liver Sinusoidal Endothelial Cells [LSECs], stellate and Kupffer cells) organized into a complex, 3D biomimetic of the human liver.

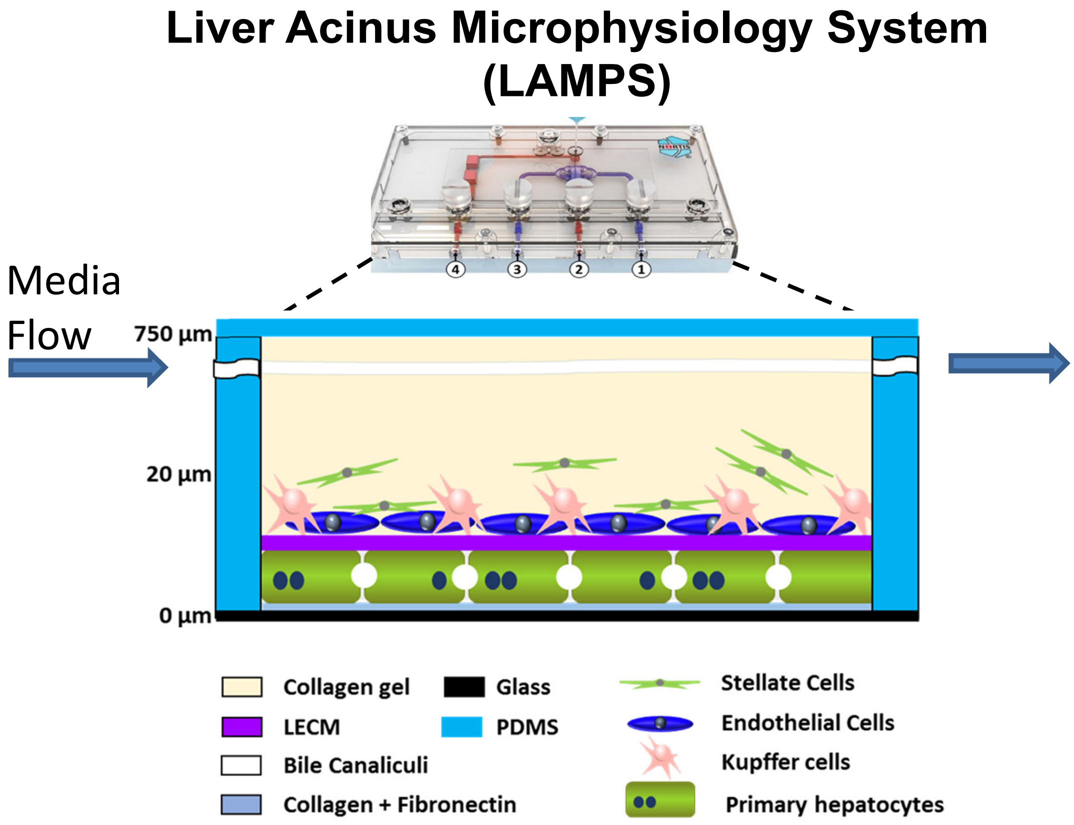

1.1.2. LipidTox neutral lipid dye staining

LipidTOX Deep Red Neutral Lipid Stain (# H34477) and Hoechst 33342 (# H3570) were obtained from ThermoFisher. 1:500 LipidTox neutral lipid dye was added into the fixed and Triton-X100 treated devices for overnight incubation at 4°C. The LipidTox dye was aspirated and the devices repetitive washed 3 times for 5 minutes in PBS and then counterstained with 6 µg/ml Hoechst for 2 hour. The devices were rinsed twice in PBS and imaged.

1.1.3. Steatosis imaging

Images were collected with a Nikon 20x (0.45 NA) objective using the INCell Analyzer 6000 (GE Healthcare) in confocal mode using the 405 nm (Hoechst) and 640 nm (LipidTOX) lasers and associated filter sets with the aperture set to 1.86 airy unit. Images for each device were acquired using the same exposure time and laser power settings. Z-stacks totaling 64 µm distance (2 µm spacing between slices) were obtained, reduced to one maximum intensity projection and then imported into FIJI (ImageJ) to quantitate steatosis (lipidTox) using interactive selection of a threshold (default method) followed by analyze particles function used circularity setting at 0.6 -1 used to measure the total intensity of the steatotic vacuoles. A total of 8 images per device were collected for analysis.

1.1.4. Secretome measurements

Efflux media from LAMPS devices was collected on days 1, 2, 4, 6, 8 and 10 to measure media levels of albumin, blood urea nitrogen, lactate dehydrogenase. The primary anti human albumin and conjugation antibody for enzyme linked immunosorbent assay (ELISA) analysis for albumin were purchased from Bethyl Laboratories (Montgomery TX). The CytoTox 96 for lactate dehydrogenase (LDH) and the urea nitrogen test were purchased from Promega (Madison, WI) and Stanbio Laboratory (Boerne, TX), respectively. HMGB-1 (Novus Biologics, Littleton, Co).

1.1.5. Multiplex immunoassays.

Efflux media was collected for each media treatment group on days 2, 6, and 10 and the levels of various human cytokines were assayed for chemokine and cytokines CCL2, IL-1B, IL-6, TNF-α, IL-8 and IL-10 from customized from previous efforts (Manush publication) using a customized Human XL Cytokine Discovery Panel (6-plex; R&D systems). Assays were completed according to the manufacturer's instructions at The University of Pittsburgh Cancer Proteomics Facility LuminexR Core Laboratory. All multiplex panels were run at the same time to avoid run-to-run and operator error variability utilizing the xMAP platform licensed by LuminexR and the results reported as pg/mL. Statistical significance was determined by two-tailed t-test. P values less than 0.05 were considered statistically significant.

1.1.6. Live Cell Readouts for Bile Efflux and Reactive Oxygen Species (ROS) Generation

Live cell readouts of LAMPS devices under flow for 10 days were used to evaluate treatment related effects on bile canalicular efflux and generation of ROS. Briefly, control or drug dosing media was aspirated from the devices and a bolus cocktail of 2 µM CMFDA (5-chloromethylfluorescein diacetate aka Cell Tracker Green, Thermo Fisher, Waltham, MA) and 5 µM dihydroethidium (DHE, Thermo Fisher, Waltham, MA) in NFM was added into each device. The chips were incubated 1 hour at 37°C. The devices were then placed on the IN Cell 6000 HCA instrument for collection of 488/525nm (ex/em) and 520/578 nm (ex/em). To reduce front to back variation during live cell image collection, 10X and 20X fields for each device were acquired using the same exposure time and laser power settings and Z-stacks setting totaling 64 µm distance (2 µm spacing between slices) were obtained, reduction of z-stacks to one maximum intensity projection which could be then imported into FIJI (ImageJ) to measure intensity of CMFDA and ROS fluorescence in the nuclear region of hepatocytes. Interactive thresholding feature in FuJi Image J was used to measure the total intensity of the CMFDA activity in the bile canaliculi between the hepatocytes. ROS intensity was determined by setting circularity to 0.6 -1 which measured just nuclear signal for DHE intensity in the hepatocytes.

1.1.7. Tocilizumab (TCZ) Binding in Live and Fixed Hepatocytes.

A 50 µL aliquot of hepatocytes at 840,000 hepatocytes/ml were plated into a 96 well clear bottom plate coated with Collagen Type 1. The hepatocytes were allowed overnight attachment and spreading. The overnight plating media was decanted and replaced with a 1.5 mg/mL solution of Collagen Type 1 prepared in hepatocyte maintenance media. The solution was allowed to gel for one hour in an incubator (37°C, 5% CO2). Fresh media was added and the overlay cultures allowed an overnight stability period. The overnight media was decanted and replaced with media containing 2.25 µg/mL biotin-anti-tocilizumab for 2-hour incubation. The drug media was decanted and the plate cultured rinsed 3 times in media to remove unbound conjugated drug. The last media rinse was decanted and replaced with 1:1000 streptavidin Cy5/6 µg/mL Hoechst nuclear dye in media for 60 minutes. The plate was rinsed 3 X in media fresh. The final media rinse was not decanted during the live cell image collection on the VTI. Media from the plate was then decanted and replaced with 4% paraformaldehyde for 30 minutes. The formaldehyde was decanted, replaced with PBS and the post fixation plate read once more on the VTI.

1.1.8. Mass spectrometry measurements

Hepatocyte maintenance media supplemented with 1 μM terfenadine was perfused through a test device on day 10 and collected for 24 hours. Terfenadine is metabolized through CYP3A4 into the double hydroxylated product, fexofenadine. The parent drug and metabolite were extracted from collected efflux media by added 2X bolus of acetonitrile, vigorous vortexing for 30 seconds, 21,000 rpm X 5 minutes in Eppendorf minifudge to pellet insoluble materials. A 20 μL aliquot of supernatant from acetonitrile extractions was added to 180 μL of 20/80 acetonitrile/H_2O (v/v) and submitted to the Mass Spectroscopy core at University of Pittsburgh for measurement. The concentration of terfenadine and fexofenadine was determined using a Waters Acquity UPLC (Milford, MA) using a C18, 1.7 μm, 2.1 X 100 mm reversed-phase column. Detection and quantitation were achieved in the positive ion mode with a TSQ Quantum Ultra Mass Spectrometer (ThermoFisher, Pittsburgh, PA), interfaced via an electrospray ionization (ESI) probe.

1.1.9. Metabolomics analysis

Sample preparation for metabolomic profiling was carried out at Metabolon Inc. Briefly, individual samples were subjected to methanol extraction then split into aliquots for analysis by ultrahigh performance liquid chromatography/mass spectrometry (UHPLC/MS). The global biochemical profiling analysis comprised of four unique arms consisting of reverse phase chromatography positive ionization methods optimized for hydrophilic compounds (LC/MS Pos Polar) and hydrophobic compounds (LC/MS Pos Lipid), reverse phase chromatography with negative ionization conditions (LC/MS Neg), as well as a HILIC chromatography method coupled to negative (LC/MS Polar) [1]. All of the methods alternated between full scan MS and data dependent MSn scans. The scan range varied slightly between methods but generally covered 70–1000 m/z.

Metabolites were identified by automated comparison of the ion features in the experimental samples to a reference library of chemical standard entries that included retention time, molecular weight (m/z), preferred adducts, and in-source fragments as well as associated MS spectra and curated by visual inspection for quality control using software developed at Metabolon. Identification of known chemical entities was based on comparison to metabolomic library entries of purified standards[2].

1.1.10. Statistical Analysis

Standard statistical significance analyses were performed in Jupyter notebook on log-transformed data. For analyses not standard in Jupyter Notebook, OmicsSoft Studio and the R program (http://cran.r-project.org/) were used. Following log transformation and imputation of missing values, if any, with the minimum observed value for each compound, 2-way ANOVA was used as significance test to identify biochemicals that differed significantly ($p < 0.05$) between experimental groups. An estimate of the false discovery rate (q-value) was calculated to take into account the multiple comparisons that normally occur in metabolomic-based studies. For the scaled intensity graphics, each biochemical in the study (raw peak area count) was rescaled to set the median across all samples equal to 1.

1.2. Development of BIOLOGXsym

BIOLOGXsym is a mechanistic, mathematical model of biologics-induced liver injury (BILI), which is organized into various sub-models that represent physiological processes involved in BILI (Figure S2A). These sub-models are mathematically integrated to simulate responses in the intact organism. Key sub-models within BIOLOGXsym are described in this section.

1.2.1. Oxidative stress sub-model

The balance of reactive nitrogen/oxygen species (RNS/ROS) was represented by zero-order RNS/ROS production and saturable, first-order clearance (Figure S2B). RNS/ROS production can be further enhanced by xenobiotics, which was represented as a first-order, non-linear process. All simulated compounds marked as RNS/ROS inducers in the mechanism selector can increase the amount of RNS/ROS in the liver.

1.2.2. Bile acid homeostasis sub-model

The bile acid homeostasis sub-model represents the synthesis and metabolism of bile acids in hepatocytes, the transporter-mediated biliary excretion and basolateral efflux of bile acids, synthesis of secondary bile acids and deconjugation of conjugated bile acids in the intestinal lumen, reabsorption of bile acids from the intestinal lumen, transporter-mediated hepatic uptake of bile acids, and the FXR-mediate feedback regulation of bile acid metabolism and transport (Figure S2C). CDCA, LCA, and respective metabolites were explicitly represented whereas the rest of bile acids are lumped as "bulk" bile acids. Accumulation of bile acids in hepatocytes can lead to mitochondrial dysfunction and decline of ATP synthesis. Details about the bile acid homeostasis sub-model were previously published.[3,4]

1.2.3. Cellular ATP sub-model

Basal ATP turnover was represented with a simple production and utilization balance (Figure S2D). The steady-state ATP turnover rate was calculated based on the measured whole body basal metabolic rate, the fraction of the basal metabolic rate from the liver, the mass of the liver, and the weighted average of the energetic cost of synthesizing ATP from fatty acids, carbohydrates, and amino acids.[5–10] Hepatic accumulation of bile acids and increased ROS/RNS can lead to reductions in ATP production. Disruptions in ATP synthesis can lead to alterations in other cellular processes, and necrosis can occur when hepatic ATP levels are significantly reduced.

1.2.4. Hepatocyte life-cycle sub-model

The hepatocyte life-cycle sub-model represents mature, mitotic, and young hepatocytes (Figure S2E). Hepatocytes can undergo death by apoptosis or necrosis, which is driven by cell stress. The energy state of the hepatocytes is an important factor that determines the type of cell death. Apoptosis can be initiated by mitochondrial and extra-mitochondrial signals and requires sufficient cellular energy in the form of ATP. Necrosis can occur when ATP levels are substantially reduced. An increased level of oxidative species can also lead to hepatocyte necrosis by compromising the integrity of the plasma membrane. Details about the hepatocyte life-cycle sub-model were previously published.[11,12]

1.2.5. Biomarker sub-models

Several clinical biomarkers were included in the model including alanine aminotransferase (ALT, Figures S1F), aspartate aminotransferase (AST), prothrombin time, total plasma bilirubin (Figures S2G), keratin 18, and high mobility box group 1 protein (HMGB1). Details about the construction of the biomarker sub-model are described in other publications.[11–14]

1.2.6. IL-6 sub-model

The IL-6 sub-model includes explicit representations of IL-6, sIL-6R, and sIL-6R complex. Formation of the sIL-6R complex is determined based on the plasma concentrations of free IL-6 and sIL-6R and binding rate data.[15] The simulated healthy individual has steady state concentrations of 3.76 pg/mL IL-6 and 40000 pg/mL sIL-6R.[16] The simulated individual with elevated IL-6 levels, representative of IL-6 levels seen in rheumatoid arthritis patients, has 100-fold more IL-6 at steady state than the healthy individual. Production of IL-6, sIL-6R, and the IL6-sIL6R complex were implemented as follows:

$$\frac{dIL6}{dt} = \Big(\big(IL6prod_{base} + IL6prod_{Mac} * (TotalMacs) + IL6prod_{LSEC} * (TotalLSEC) + IL6prod_{neut} * (TotalNeuts) + IL6prod_{HC} * (TotalMatureHCs)\big)/V_{plasma}\Big) - IL6sIL6R_{bind} * (1/sIL6R_{MW}) * IL6 * sIL6R_{eff} + IL6sIL6R_{dissoc} * \big(IL6_{MW}/(IL6_{MW} + sIL6R_{MW})\big) * Complex_{IL6-sIL6R} - IL6 * \big(log(2)/IL6_{1/2}\big)$$

$$\frac{dsIL6R}{dt} = sIL6Rprod_{base}/V_{plasma} - IL6sIL6R_{bind} * (1/IL6_{MW}) * IL6 * sIL6R_{eff} + IL6sIL6R_{dissoc} * \big(sIL6R_{MW}/(IL6_{MW} + sIL6R_{MW})\big) * Complex_{IL6-sIL6R} - sIL6R_{eff} * \big(log(2)/sIL6R_{1/2}\big)$$

$$\frac{dComplex_{IL6-sIL6R}}{dt} = IL6sIL6R_{bind} * \big((IL6_{MW} + sIL6R_{MW})/(IL6_{MW} * sIL6R_{MW})\big) * IL6 * sIL6R_{eff} - IL6sIL6R_{dissoc} * Complex_{IL6-sIL6R} - Complex_{IL6-sIL6R} * \big(log(2)/Complex_{1/2}\big)$$

$$IL6_{Eff} = IL6 * (1 - mIL6R_{Block})$$

$$sIL6R_{eff} = sIL6R * (1 - sIL6R_{Block})$$

where prod indicates a production rate, base indicates the basal level, Mac indicates macrophages, LSEC indicates liver sinusoidal endothelial cells, neut indicates neutrophils, HC indicates hepatocytes, and V_{plasma} is the volume of plasma. Formation of the signaling complex is then characterized by a binding rate ($IL6sIL6R_{bind}$) and a dissociation rate ($IL6sIL6R_{dissoc}$). Each species has a molecular weight (MW) and a half-life (1/2). The effective concentration of sIL-6R is given by $sIL6R_{eff}$.

Key effects of IL-6 signaling on hepatocytes and relevant biomarkers were included in the sub-model. Included effects were determined by availability of data to support IL-6-mediated contributions and parameterization of effect in addition to consideration of effects most likely to influence liver environment, health, and biomarkers. Signaling via soluble IL-6R drives upregulation of macrophage recruitment to the liver and hepatocyte regeneration (Figures S2H).[16–22] IL-6 signaling through both membrane-bound and soluble-IL-6R drives suppression of CYP activity (CYP2E1, CYP3A4) and upregulation of plasma CRP levels (Figures S2H).[23–29] Parameterization of IL-6 dependent changes in CYP activity was optimized based on LAMPS and literature data.[27,30] Parameterization of change in CRP production was optimized based on literature data.[24] Effect of IL-6 signaling on hepatocyte proliferation and macrophage recruitment was optimized to be at a similar magnitude to the other contributing mediators. Downstream effects of IL-6 signaling are implemented as follows:

$$CYP_{signal} = max\Big(0, IL6_{CYPContrib} * \big((IL6_{DelayEffect} + Complex_{DelayEffect})/(IL6_{NHVo} + Complex_{NHVo})\big)\Big)$$

$$\mathrm{CYP1}_{prod} = \mathrm{CYP1}_{Emax} - (CYP1_{Emax} - CYP1_{Emin}) * \Big(CYP_{signal}^{CYP1_{Hill}}\Big)/\Big(CYP1_{EC50}^{CYP1_{Hill}} + CYP_{signal}^{CYP1_{Hill}}\Big)$$

$$\frac{dCRP}{dt} = \Big(\big(CRPprod_{base} + CRPprod_{HC} * (TotalMatureHCs)\big)/V_{plasma}\Big) - CRP * \big(log(2)/CRP_{1/2}\big);$$

$$CRP_{signal} = max\Big(0, IL6_{CRPContrib} * \big((IL6_{DelayEffect} + Complex_{DelayEffect})/(IL6_{NHVo} + Complex_{NHVo})\big) - 1\Big)$$

$$\mathrm{CRPprod}_{HC} = \mathrm{CRP}_{Vmax} * \Big(CRP_{signal}^{CRP_{Hill}}\Big)/\Big(CRP_{Km}^{CRP_{Hill}} + CRP_{signal}^{CRP_{Hill}}\Big)$$

$$Mac_{RecruitSignal} = max\Bigg(0, \bigg(\Big((IL10o + IL10_{inhibMac})/\big(IL10_{DelayEffect} + IL10_{inhibMac}\big)\Big) * \Big(\big(TNF_{DelayEffect}/TNFo - 1\big) + \big(DAMPs_{DelayEffect}/DAMPs_o - 1\big) + Complex_{contribMac} * (Complex_{IL6-sIL6R}/Complex_{NHVo} - 1)\Big)\bigg)\Bigg)$$

$$\mathrm{H}C_{\mathrm{Prolif}\mathrm{Signal}} = \max\Big(0, HGF_{ProlifCoeff} * \big(HGF_{DelayEffect}/HGF_o - 1\big) + TNF_{ProlifCoeff} * \big(TNF_{DelayEffect}/TNF_o - 1\big) + IL6sIL6R_{ProlifCoeff} * \big(Complex_{DelayEffect}/Complex_{NHVo} - 1\big)\Big)$$

where CYP_{signal} represents the signal received by IL-6 signaling, CYPContrib represents the scale of the effect IL-6 has on CYP signaling, DelayEffect indicates the concentration of the species including a delay between initial signal and response for membrane- and soluble- IL-6R signaling, and NHVo indicates the healthy baseline concentration for that species. The CYP production rate for a given CYP (e.g., $CYP1_{prod}$) is then determined as a hill function with the signal as input and CYP-specific parameters $CYP1_{Emax}$, $CYP1_{Emin}$, $CYP1_{Hill}$, and $CYP1_{EC50}$ representing the max expression, the minimum expression, the hill number, and the EC50 for that CYP respectively.

Plasma CRP levels include basal production, production by hepatocytes controlled by IL-6 signaling, and a half-life, with terms defined as above.

The recruitment signal for macrophages includes IL-6 signaling via sIL-6R only. The contribution of IL-6 signaling to the recruitment signal relative to the other mediators (IL-10, TNF-α, and DAMPs) is given by $Complex_{contribMac}$. Macrophage recruitment rate is then calculated as a hill function of the signal, as described previously.[31]

Based on the supporting literature, only IL-6 signaling through the soluble IL-6R is included as contributing to the hepatocyte proliferation signal ($HC_{ProlifSignal}$). This is in addition to signals from HGF and TNF-α, with relative contribution of IL-6 signaling defined by $IL6sIL6R_{ProlifCoeff}$. The hepatocyte proliferation rate is then calculated as a hill function of the signal as described previously.[11,12]

Tocilizumab inhibits IL-6 signaling by binding to both the membrane-bound and soluble IL-6 receptor. This mechanism is included in BIOLOGXsym as follows:

$$\mathrm{sIL6R}_{\mathrm{blockade}Effect} = \mathrm{sIL6R}_{\mathrm{blockadeVmax}} * \left(\mathrm{plasma}_{\mathrm{TCZ}}^{\mathrm{sIL6R_{blockadeHill}}} / \left(\mathrm{sIL6}R_{blockadeKm}^{sIL6R_{blockadeHill}} + \mathrm{plasma}_{TCZ}^{sIL6R_{blockadeHill}}\right)\right)$$

$$\mathrm{mIL6R}_{\mathrm{blockade}Effect} = \mathrm{mIL6R}_{\mathrm{blockadeVmax}} * \left(\mathrm{LiverInterstitial}_{\mathrm{TCZ}}^{\mathrm{mIL6R_{blockadeHill}}} / \left(\mathrm{mIL6}R_{blockadeKm}^{mIL6R_{blockadeHill}} + \mathrm{LiverInterstitial}_{TCZ}^{mIL6R_{blockadeHill}}\right)\right)$$

$$sIL6R_{eff} = sIL6R * \left(1 - sIL6R_{blockadeEffect}\right)$$

$$\mathrm{IL6}_{eff} = \mathrm{IL6} * \left(1 - mIL6R_{blockadeEffect}\right)$$

where the effect of TCZ on sIl6R and mIL6R signaling ($sIL6R_{blockadeEffect}$, $mIL6R_{blockadeEffect}$ respectively) is represented by hill equations with the concentration of plasma TCZ and liver interstitial TCZ as inputs respectively. Inhibition of signaling via sIL-6R is represented as a decrease in the concentration of free sIL-6R ($sIL6R_{eff}$) available to form IL6-sIL6R complex. Inhibition of signaling via membrane-bound IL-6R is represented implicitly as a reduction in the concentration of effective IL-6 ($IL6_{eff}$) controlling membrane-bound receptor signaling targets including CRP levels and CYP activity.

Impact of biologics on hepatic transporter function

The impact of biologics on hepatic transport of bile acid and bilirubin is represented as indirect effects based on hepatic interstitial exposure of biologics as follows:

$$\frac{dDelay1, BiolPathway_{BA,Bili_{CL,ML,PP}}}{dt} = Tau, BiolPathway_{BA,Bili} * (InterstialBiol_{CL,ML,PP} - Delay1, BiolPathway_{BA,Bili_{CL,ML,PP}})$$

$$\frac{dDelay2, BiolPathway_{BA,Bili_{CL,ML,PP}}}{dt} = Tau, BiolPathway_{BA,Bili} * (Delay1, BiolPathway_{BA,Bili_{CL,ML,PP}} - Delay2, BiolPathway_{BA,Bili_{CL,ML,PP}})$$

$$\frac{dDelay3, BiolPathway_{BA,Bili_{CL,ML,PP}}}{dt} = Tau, BiolPathway_{BA,Bili} * (Delay2, BiolPathway_{BA,Bili_{CL,ML,PP}} - Delay3, BiolPathway_{BA,Bili_{CL,ML,PP}})$$

$$Signal, BiolPathway_{BA,Bili_{CL,ML,PP}} = 1 + \frac{V_{max}, BiolPathway_{BA,Bili} * Delay3, BiolPathway_{BA,Bili_{CL,ML,PP}}^{Hill,BiolPathway_{BA,Bili}}}{K_m, BiolPathway_{BA,Bili}^{Hill,BiolPathway_{BA,Bili}} + Delay3, BiolPathway_{BA,Bili_{CL,ML,PP}}^{Hill,BiolPathway_{BA,Bili}}}$$

$$ModulatedPathway_{BA,Bili_{CL,ML,PP}} = Signal, BiolPathway_{BA,Bili_{CL,ML,PP}} * BasalPathway_{BA,Bili_{CL,ML,PP}}$$

in which $BiolPathway_{BA,Bili}$ = a bile acid- or bilirubin-related disposition pathway impacted by biologic present in the hepatic interstitium; $\frac{dDelay1-3}{dt}$ = rate of change in delay compartments 1-3 to accommodate an empirical, transcription-like effect; CL, ML, PP = centrilobular (CL), midlobular (ML) or periportal (PP) zones of the liver; Tau = delay parameter; $InterstialBiol$ = concentration of biologic in the hepatic interstitium; $Signal$ = a transcriptional signal mediated by biologic in the hepatic interstitium; V_{max} = the maximum velocity in a Michaelis-Menten-type relationship ranging from -1 to ∞ to generate the transcriptional signal; $Hill$ = the hill coefficient in a Michaelis-Menten-type relationship to generate the transcriptional signal; K_m = the concentration in delay compartment 3 that yields half of the maximum velocity in a Michaelis-Menten-type relationship to

generate the transcriptional signal; $ModulatedPathway$ = the transcriptionally modulated bile acid- or bilirubin-related disposition pathway; $BasalPathway =$ the basal bile acid- or bilirubin-related disposition pathway.

Figure S2. Diagrams of key sub-models within BIOLOGXsym. **A**) Diagram of the overall structure of BIOLOGXsym. B) Oxidative stress sub-model. **C**) Bile acid homeostasis sub-model. **D**) Cellular ATP sub-model. **E**) Hepatocyte life-cycle sub-model. **F**) ALT sub-model. **G**) Bilirubin sub-model. **H**) IL-6 sub-model.

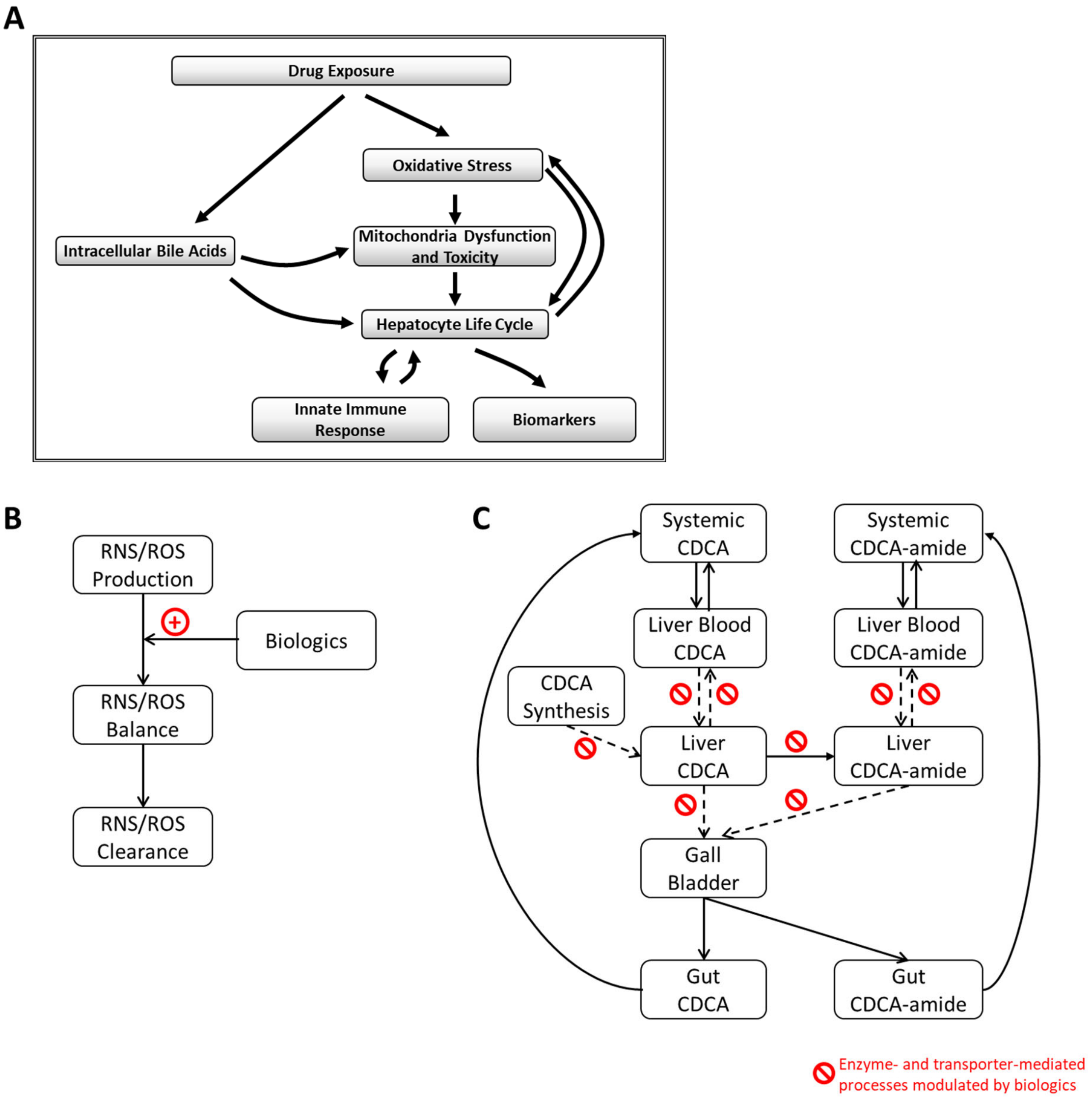

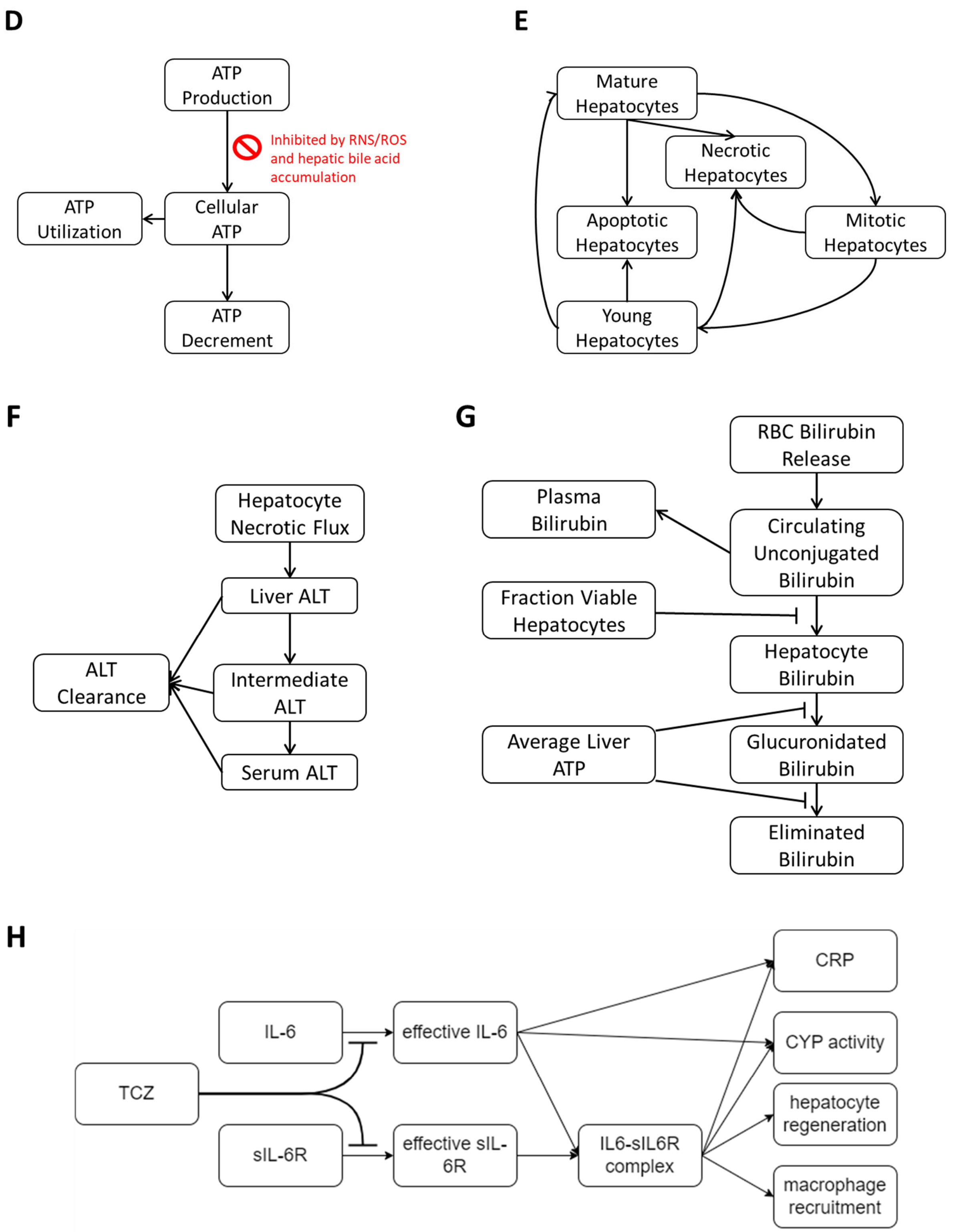

1.3. Physiologically-based pharmacokinetic (PBPK) modeling of tocilizumab (TCZ)

A PBPK model of TCZ was developed using the biologics module of GastroPlus® 9.8. The model included clearance of TCZ through membrane-bound IL-6 receptor (mIL-6R). It was assumed that the concentration of TCZ is significantly greater than the concentration of soluble IL-6 receptor (sIL-6R) in the plasma. This assumption was supported by data indicating that sIL-6R is more than 95% bound for the duration of TCZ exposure and that the relative molarity of TCZ

and sIL-6R indicated a TCZ concentration approximately 10-fold higher than sIL-6R concentration [32]. Based on the relative concentration of TCZ and sIL-6R, it was assumed that sIL-6R did not significantly affect the concentration of free TCZ.

To parameterize the model, available data from the literature for TCZ binding to sIL-6R [33], TCZ binding to FcRn [34], and TCZ-mIL6R internalization [34] were used. It was assumed that the binding rate of TCZ to mIL-6R was similar to binding sIL-6R. Relative antigen expression levels across organs were obtained from a published literature [34] and absolute expression levels were scaled to match plasma TCZ clearance. The degradation rate of free TCZ was optimized to fit plasma TCZ data. The PBPK model was optimized to a single dose of 81 mg and 162 mg intravenous and subcutaneous data in normal healthy volunteers [35]. Model was validated on repeat dosing data for 162 mg subcutaneous TCZ dosed once per week or twice per week in rheumatoid arthritis patients [36]. This model was used to predict the hepatic interstitial concentration of free TCZ for a healthy 91 kg male with BMI of 29 kg/m^2 (to match the average body weight for the BIOLOGXsym SimCohorts) administered 8 mg/kg IV TCZ Q4W for twelve weeks. Drug-specific parameters, attained from the literature or estimated using available plasma TCZ concentration versus time data, are listed in Table S1.

Table S1. Key TCZ parameters used in GastroPlus simulations.

Parameter	Value	Units	Source
Molecular Weight	145000	g/mol	[37]
TCZ-mIL-6R Kon	19008	1/µM/d	[33]
TCZ-mIL-6R Koff	95.04	1/day	[33]
TCZ-mIL-6R Kint,TMD	96.048	1/day	[34]
mIL-6R Kdeg	6.6528	1/day	[34]
Fvv	0.01	dimensionless	Optimized to clinical exposure data
Lung Ag expression	2.1e-6	µmol/g tissue	Adapted from [34]
Liver Ag expression	3.07e-6	µmol/g tissue	Adapted from [34]
Heart Ag expression	4.38e-6	µmol/g tissue	Adapted from [34]
Kidney Ag expression	9e-7	µmol/g tissue	Adapted from [34]
Spleen Ag expression	6.1e-5	µmol/g tissue	Adapted from [34]
Muscle Ag expression	5e-6	µmol/g tissue	Adapted from [34]
Skin Ag expression	3.33e-4	µmol/g tissue	Adapted from [34]
Vascular reflection coeff	0.95	dimensionless	GastroPlus default
Lymphatic reflection coeff	0.2	dimensionless	GastroPlus default
Exog endosomal uptake rate	6	1/day	Optimized to clinical exposure data
Recycle rate	12	1/day	GastroPlus default
Vascular rate fraction	0.72	dimensionless	GastroPlus default
Kon, FcRn(7.4)	0	1/µM/d	[34]
Kon, FcRn (6.0)	1.901e4	1/µM/d	[34]
Koff, FcRn (7.4)	0	1/day	[34]

Koff, FcRn (6.0)	3974.4	1/day	[34]
Kdeg	10	1/day	Optimized to clinical exposure data

1.4. PBPK modeling of GGF2

To simulate *in vivo* exposure of GGF2, a PBPK model of GGF2 was developed using the biologics module within GastroPlus 9.8. Since naturally occurring neuregulin (NRG)-1β binds to erythroblastic leukemia viral oncogene (ErbB) receptors 3 and 4, which homodimerize and/or heterodimerize with the ErbB2 receptor [38,39], these receptors were considered for describing the non-linear target-mediated drug disposition of recombinant NRG-1β (*i.e.*, GGF2). Internalization rate constants for GGF2-bound ErbB3 and ErbB4 in the model were estimated based on the reported constant for antibody-bound epidermal growth factor receptor (EGFR/ErbB1) [34], and the receptor half-lives of EGF-bound EGFR, and EGF-bound EGFR/ErbB-3 and EGFR/ErbB-4 chimeras [40]. Degradation rate constants for free ErbB3 and ErbB4 were derived from the same study [40]. Interstitial tissue concentrations of EGFR [34] in conjunction with organ- and cell type-specific protein expression data of EGFR and ErbB3-4 from the Human Protein Atlas (immunohistochemically annotated as "not detected", "low", "medium" or "high") were used to estimate interstitial tissue concentrations of ErbB3-4 in heart, liver, lung, spleen, kidney, skin, and muscle. Furthermore, since the GGF2 glycoprotein is a relatively small biologic (52.6 kDa), parameters governing convection- and diffusion-related processes were optimized in GastroPlus based on preclinical and clinical data of GGF2 [38,39,41]. Drug-specific parameters, attained from the literature or estimated using available plasma GGF2 concentration data, are listed in Table S2.

Table S2. Key GGF2 parameters used in GastroPlus simulations.

**Parameter**	**Value**	**Units**	**Source**
Molecular weight	52600	g/mol	Parry 2017
Vascular reflection coeff	0.5	dimensionless	Optimized to preclinical and clinical data[38,39,41]
Lymphatic reflection coeff	0.5	dimensionless	Optimized to preclinical and clinical data[38,39,41]
Endosomal uptake rate	12	1/day	Optimized to preclinical and clinical data[38,39,41]
Recycle rate	12	1/day	Optimized to preclinical and clinical data[38,39,41]
Vascular Rate Fraction	0.72	dimensionless	Optimized to preclinical and clinical data[38,39,41]
Kon,FcRn(7.4)	0	1/µM/day	No association between FcRn and GGF2 assumed
Kon,FcRn(6.0)	0	1/µM/day	No association between FcRn and GGF2 assumed
Koff,FcRn(7.4)	3.0E+4	1/day	Default value, but no association between FcRn and GGF2 assumed
Koff,FcRn(6.0)	500	1/day	Default value, but no association between FcRn and GGF2 assumed
Kdeg	1.0E+4	1/day	Default value

ErbB3 Kdeg	4.75	1/day	Estimated using literature data[34,40]
ErbB4 Kdeg	2.77	1/day	Estimated using literature data[34,40]
ErbB3 Kon	1000	1/μM/day	Optimized to preclinical and clinical data[38,39,41]
ErbB3 Koff	10	1/day	Optimized to preclinical and clinical data[38,39,41]
ErbB3 Kint,TMD	103.68	1/day	Estimated using literature data[34,40]
ErbB4 Kon	1000	1/μM/day	Optimized to preclinical and clinical data[38,39,41]
ErbB4 Koff	10	1/day	Optimized to preclinical and clinical data[38,39,41]
ErbB4 Kint,TMD	56.55	1/day	Estimated using literature data[34,40]
Liver ErbB3 expression	1.23E-4	μmol/g tissue	Adapted from literature data[34] and the Human Protein Atlas
Liver ErbB4 expression	6.13E-5	μmol/g tissue	Adapted from literature data[34] and the Human Protein Atlas
Lung ErbB3 expression	1.71E-3	μmol/g tissue	Adapted from literature data[34] and the Human Protein Atlas
Lung ErbB4 expression	1.71E-3	μmol/g tissue	Adapted from literature data[34] and the Human Protein Atlas
Spleen ErbB3 expression	4.46E-5	μmol/g tissue	Adapted from literature data[34] and the Human Protein Atlas
Muscle ErbB3 expression	2.02E-4	μmol/g tissue	Adapted from literature data[34] and the Human Protein Atlas
Heart ErbB3 expression	2.63E-4	μmol/g tissue	Adapted from literature data[34] and the Human Protein Atlas
Kidney ErbB3 expression	1.86E-4	μmol/g tissue	Adapted from literature data[34] and the Human Protein Atlas
Kidney ErbB4 expression	6.20E-5	μmol/g tissue	Adapted from literature data[34] and the Human Protein Atlas
Skin ErbB3 expression	9.98E-3	μmol/g tissue	Adapted from literature data[34] and the Human Protein Atlas
Skin ErbB4 expression	6.65E-3	μmol/g tissue	Adapted from literature data[34] and the Human Protein Atlas
Organ-specific lymph flow	100	(% of plasma flow)	Optimized to preclinical and clinical data[38,39,41], to account for other potential diffusion processes

2. RESULTS

2.1. Evaluation of TCZ and GGF2 in LAMPS

Albumin and urea responses were measured in LAMPS models treated with TCZ (232 µg/mL and 725 µg/mL), IL-6 (3 ng/mL), combination of TCZ (232 µg/mL) and IL-6 (3 ng/mL), or GGF2 (10, 100, and 382 ng/mL) (Figure S3). Albumin and urea responses were not significantly different from controls for 232 µg/mL and 725 µg/mL Tocilizumab, 3 ng/mL IL-6, or combination of TCZ (232 µg/mL) and IL-6 (3 ng/mL) (Figure S3A, C). Albumin and urea responses were not significantly different from controls for 10, 100, and 382 ng/mL GGF2 groups (Figure S3B, D). Efflux of glycochenodeoxycholic acid (GCDCA) and taurocholic acid (TCA) was not altered by tocilizumab (232 µg/mL and 725 µg/mL) (Figure S4).

Figure S3. Albumin (**A, C**) and urea (**B, D**) measurements on day 10 in LAMPS chips treated with 1.6 µM (232 µg/mL) or 5 µM (725 µg/mL) TCZ, IL-6 (3 ng/mL), combination of TCZ (232 µg/mL) and IL-6 (3 ng/mL), or GGF2 (10, 100, and 382 ng/mL). N = 15, 9, 3, 6, 6, 4, 4, 3 chips for control, 232 µg/mL TCZ, 725 µg/ml TCZ IL-6, IL-6 + TCZ, 10 ng/mL GGF2, 100 ng/mL GGF2, and 382 ng/mL GGF2 groups, respectively. Values presented as mean ± SEM.

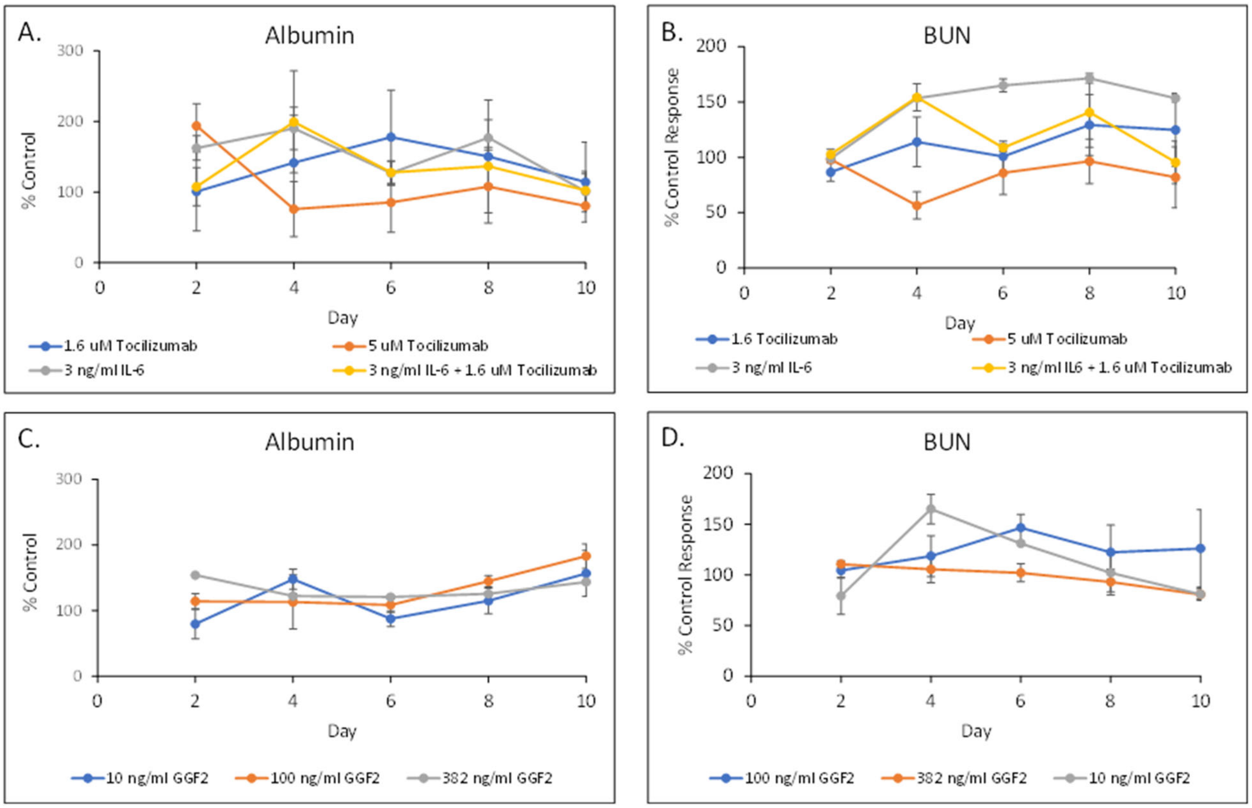

Figure S4. Efflux of glycochenodeoxycholic acid (GCDCA) and taurocholic acid (TCA) was not altered by tocilizumab.

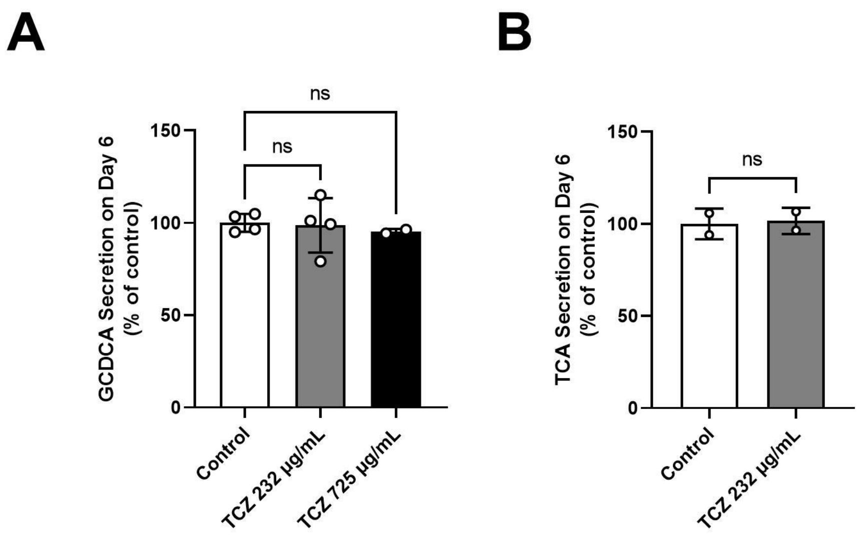

2.2. PBPK modeling of TCZ

Simulated plasma TCZ exposure was generally in agreement with the reported clinical pharmacokinetic data (Figure S5). For the datasets used for model optimization, simulated $AUC_{0-480hr}$ and C_{max} for 81 mg and 162 mg TCZ administered intravenously and subcutaneously were within 0.79-2.1-fold and 0.8-1.3-fold of the observed data respectively (Figure S5) [35]. For the datasets used for model validation, the first dose and steady state AUC and C_{max} for 162 mg TCZ administered subcutaneously once per week or once per two weeks for 24 weeks were within 0.8-2.1-fold and 0.8-1.3-fold of the observed data respectively (Figure S6)[36]. The final model was used to predict plasma and liver interstitial TCZ concentrations for 8 mg/kg TCZ administered intravenously every four weeks to a 91 kg healthy male (Figure S7). The twelve-week plasma AUC and C_{max} were 80799 ug*h/mL and 177.6 ug/mL respectively. The twelve-week liver interstitial TCZ AUC and C_{max} were 16355 ug*h/mL and 36.4 ug/mL respectively. The simulated plasma and liver interstitial concentration-vs-time profiles for were then used in BIOLOGXsym to drive TCZ-mediated effects on the liver.

Figure S5. PBPK model of TCZ vs clinical data used for optimization. Simulated concentration of TCZ in the plasma after a single dose of (**A**) 81 mg IV, (**B**) 162 mg IV, (**C**) 81 mg sc, and (**D**) 162 mg sc (blue solid line) compared to clinical data (blue squares[35]). (**E**) Summary descriptors (AUC, C_{max}, t_{max}) for observed and simulated PBPK profiles and ratio of simulated to observed for each descriptor. Observed values for AUC, C_{max} represent arithmetic mean ± standard deviation. Observed values for t_{max} represent median (range).

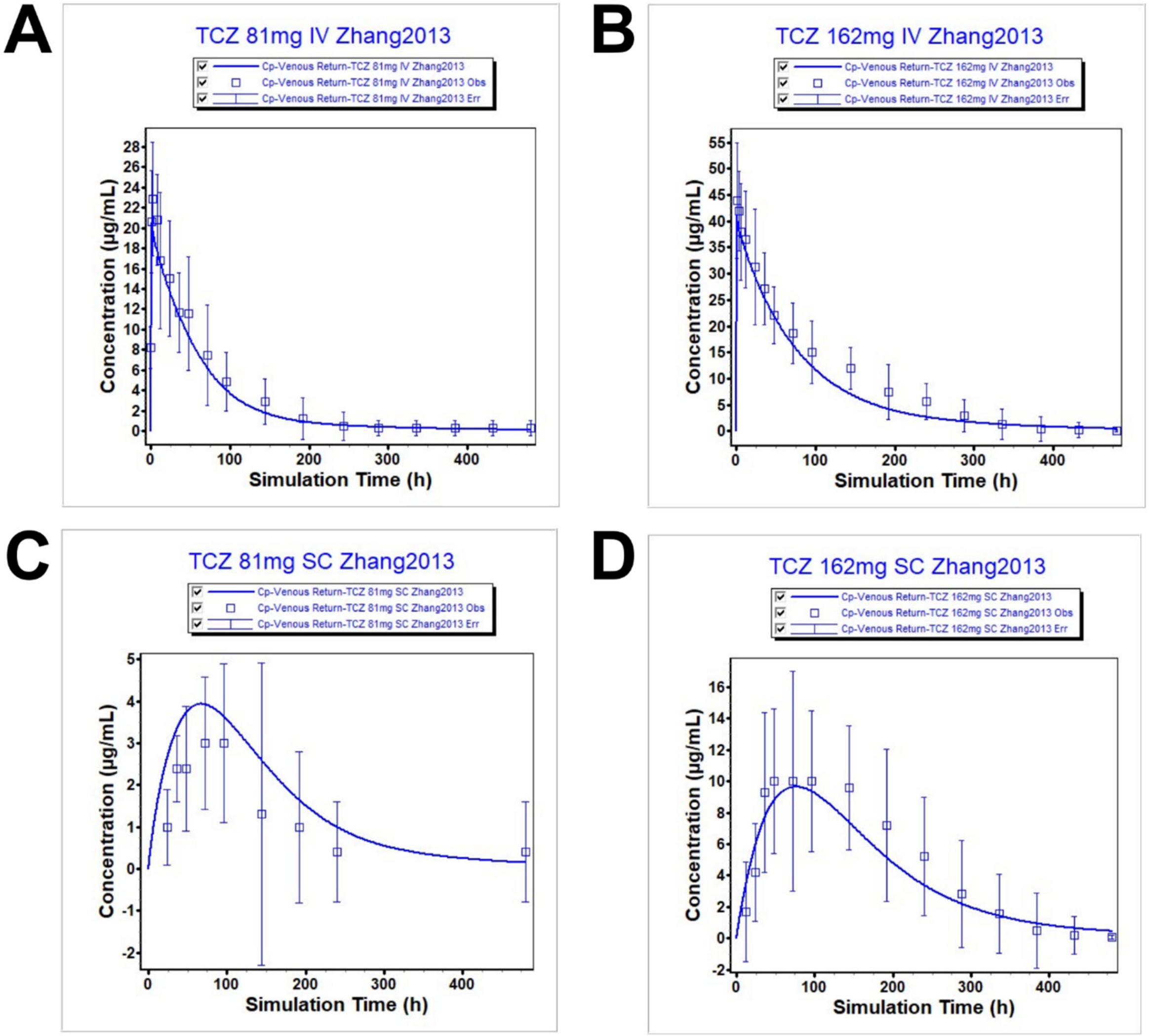

E

	TCZ iv 81 mg			TCZ iv 162 mg			TCZ sc 81 mg			TCZ sc 162 mg		
PK parameter	Obs (n=12)	Sim	Sim/Obs	Obs (n=12)	Sim	Sim/Obs	Obs (n=12)	Sim	Sim/Obs	Obs (n=12)	Sim	Sim/Obs
AUC_{last}, µgxh/mL	1390 ± 573	1270	**0.91**	4300 ± 1360	3389	**0.79**	336 ± 255	711	**2.1**	2360 ± 1240	1958	**0.8**
C_{max}, µg/mL	25.0 ± 5.3	20.5	**0.82**	47.0 ± 11.4	41.0	**0.87**	3.020 ± 2.05	3.9	**1.3**	12.4 ± 6.6	9.6	**0.8**
t_{max}, h (range)	3 (1.0-8.0)	1	**0.33**	1.5 (1.0-4.0)	1	**0.67**	96 (36-96)	67	**0.7**	72 (36-192)	76	**1.0**

Figure S6. PBPK model of TCZ vs clinical data used for validation. Simulated concentration of TCZ in the plasma after 162 mg sc (**A**) Q2W and (**B**) QW for 24 weeks (blue solid line) compared to clinical data (blue squares[36]). (**C**) Summary descriptors (AUC, C_{max}, C_{trough}) for first dosing interval and steady state dosing interval for observed and simulated PBPK profiles and ratio of simulated to observed for each descriptor. Observed values represent mean ± standard deviation.

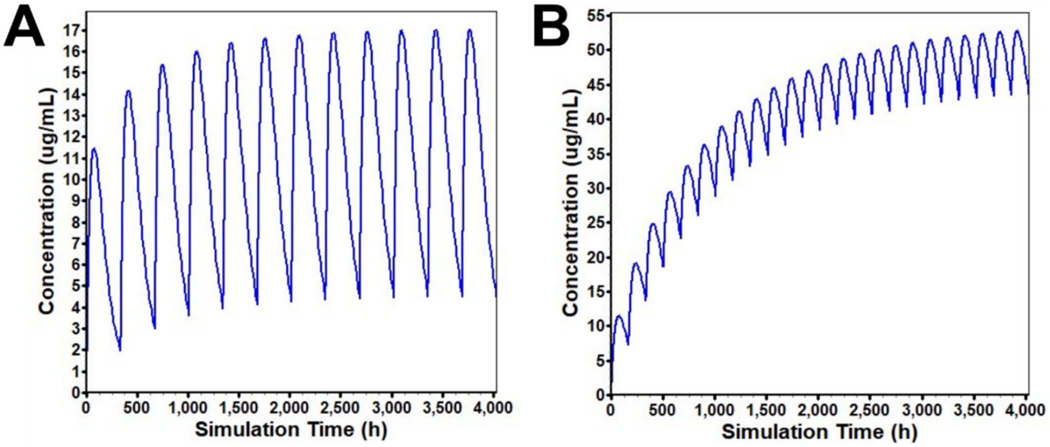

C

	TCZ sc 162 Q2W			TCZ sc 162 QW		
Parameters	Obs	Sim	Sim/Obs	Obs	Sim	Sim/Obs
1st dosing interval AUC ug h/mL	1210 ± 940	2208	**1.8**	1243 ± 689	1527	**1.23**
1st dosing interval Cmax ug/mL	5.8 ± 4.1	11.1	**1.9**	9.3 ± 5.1	11.4	**1.22**
1st dosing interval Ctrough ug/mL	1 ± 1.5	1.95	**1.95**	7.0 ± 4.1	7.3	**1.04**
SS dosing interval AUC ug h/mL	3460 ± 2530	3776	**1.1**	8254 ± 3833	8299	**1.00**
SS dosing interval Cmax ug/mL	13 ± 8.3	17.03	**1.3**	51.3 ± 23.2	52.8	**1.03**
SS dosing interval Ctrough ug/mL	5.9 ± 6.3	4.5	**0.8**	45.3 ± 22.2	43.7	**0.96**

Figure S7. Predictions from PBPK model of TCZ. Simulated concentration of TCZ in the plasma (blue) and liver interstitium (brown) for 8 mg/kg IV TCZ Q4W for twelve weeks in a 91 kg healthy adult male.

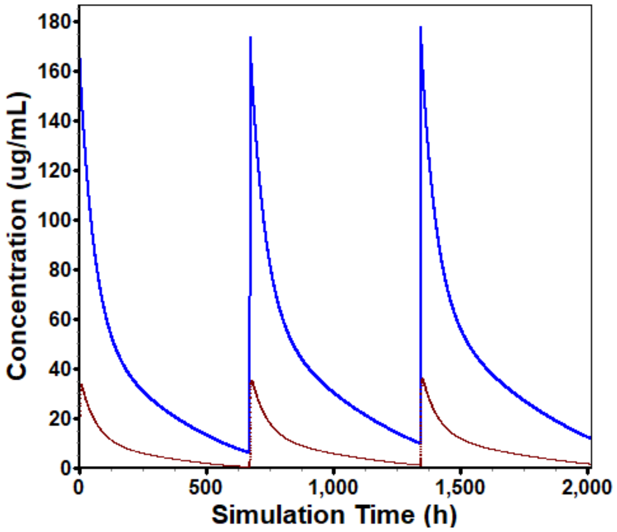

2.3. Representation of TCZ effects on IL-6 signaling

A sub-model representing IL-6 production, clearance, and binding to membrane and soluble IL-6 receptors via classic and trans-signaling pathways, respectively, were developed within BIOLOGXsym. IL-6 cross-regulation of mediators and immune cells were evaluated and added where supported by literature, including a trans-IL-6-signaling dependent increase in macrophage recruitment to the liver. Key IL-6 interactions with hepatocytes such as CRP production, hepatocyte CYP3A4 expression, and hepatocyte turn-over have been represented. LAMPS data demonstrated reduced CYP3A4 activity with increased IL-6 levels. This activity returned to baseline with added TCZ exposure. These data, along with data from the literature, were used to quantify and parameterize the change in CYP3A4 and CYP2E1 production by hepatocytes in response to changes in IL-6 signaling. An *in vitro*-like setup within BIOLOGXsym mimicking the LAMPS experiments was able to reproduce dose-dependent reduction in CYP3A4 activity in response to IL-6 increases, consistent with vLAMPS experiments and literature [30] (Figure S8A). Simulated patients with elevated IL-6 exhibited reduced expression of CYP3A4 and CYP2E1, and administration of TCZ (8 mg/kg every 4 weeks) recovered activity of these CYP isozymes (Figure S8B).

Figure S8. Effects of IL-6 and TCZ on CYP activity. **A**) IL-6 effects on CYP3A4 activity in simulations mimicking *in vitro* IL-6 exposure (red symbols) compared to experimental data (black symbols). The error bar on the observed data represents standard deviation. **B**) Simulated production of CYP3A4 and CYP2E1 in patients with elevated IL-6 and effects of TCZ administration. NHV: normal healthy volunteer.

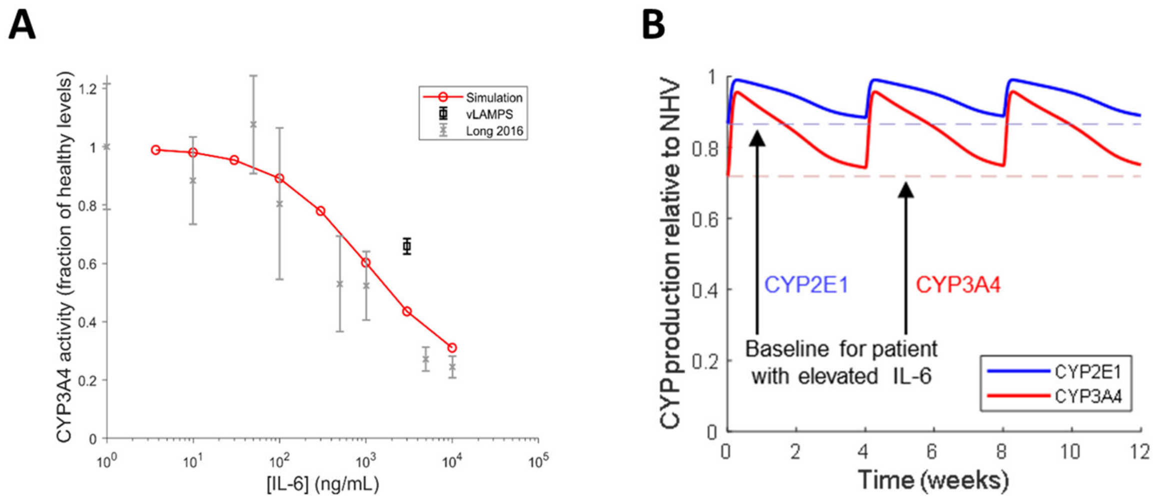

2.4. PBPK modeling of GGF2

Simulated plasma GGF2 exposure was generally in agreement with the reported clinical pharmacokinetic data. The reported, observed C_{max} in patients administered a 20-to-30-min 1.5 mg/kg intravenous bolus infusion was 0.6 µg/mL [41], but apart from "more frequent sampling on the first day" it is unclear when blood sampling for pharmacokinetic analysis was performed [38]. If no sample was taken immediately at the end of the infusion, the reported C_{max} may have been underestimated. The simulated C_{max} immediately after a 30-min infusion was 5.5 µg/mL, and plasma concentrations were predicted to reach 0.6 µg/mL in a matter of minutes (Figure S9), relatively consistent with the short half-life of GGF2 reported in rats [39]. At this dose level, the PBPK model predicted a maximum GGF2 concentration of 12 µg/mL in the hepatic interstitium. The simulated concentration-vs.-time profile of GGF2 in the hepatic interstitium was integrated in BIOLOGXsym to drive the pathophysiological effects on the liver.

Figure S9. Clinical GGF2 exposure in plasma (solid blue curve) and the hepatic interstitium (dotted maroon curve) was simulated by physiologically based pharmacokinetic modeling using GastroPlus. The red box indicates the potential timeframe post-infusion accounting for the apparent discrepancy between predicted vs. observed C_{max}, the latter of which is represented by the red, horizontal, dotted line. C_{max}, maximum plasma concentration; IV, intravenous administration

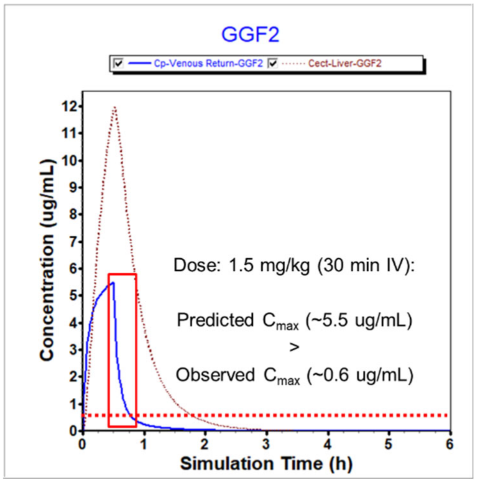

2.5. Determination of TCZ mechanistic toxicity parameters

Table S3. Mechanistic toxicity parameter values for TCZ.

**Name**	**Units**	**TCZ**
Liver RNS/ROS production rate Vmax 4	1/hour	0.03964
Liver RNS/ROS production rate Km 4	umol/L	0.2
Liver RNS/ROS production rate Hill 4	dimensionless	5
duration of VLDL release inhibition 1	hours	2500
magnitude of VLDL release inhibition 1	dimensionless	0.75
Compound Y molecular weight	g/mol	145000
mIL-6R depletion Vmax in response to CompY	ug/mL	1
mIL-6R depletion Km in response to CompY	ug/mL	1
mIL-6R depletion Hill in response to CompY	dimensionless	1
sIL-6R depletion Vmax in response to CompY	ug/mL	1
sIL-6R depletion Km in response to CompY	ug/mL	0.5
sIL-6R depletion Hill in response to CompY	dimensionless	5

2.6. Determination of GGF2 mechanistic toxicity parameters

Table S4. Mechanistic toxicity parameter values for GGF2. In addition to a V_{max} value, each of the Michaelis-Menten equations for the regulatory bile acid and bilirubin disposition mechanisms has K_M and Hill values, which were set to values of 10^{-12} μg/mL and 1, respectively, for all mechanisms. Furthermore, each pathway had a (transcription-like) delay parameter set to 0.25 hr^{-1}.

Name	Units	GGF2
Compound W molecular weight	g/mol	52600
Biologics-based LCA amidation impact Vmax for Compound W	dimensionless	-0.468
Biologics-based CDCA amidation impact Vmax for Compound W	dimensionless	-0.468
Biologics-based BA uptake impact Vmax for Compound W	dimensionless	-0.3466
Biologics-based BA basolateral efflux impact Vmax for Compound W	dimensionless	-0.68/ -0.89*
Biologics-based BA biliary efflux impact Vmax for Compound W	dimensionless	-0.39
Biologics-based OATP1B1/1B3-dependent unconjugated bilirubin uptake impact Vmax for Compound W	dimensionless	-0.55
Biologics-based conjugated bilirubin uptake impact Vmax for Compound W	dimensionless	-0.55
Biologics-based conjugated bilirubin basolateral efflux impact Vmax for Compound W	dimensionless	0.21

*A parameter value of -0.68 was able to reproduce the LAMPS data under in vitro-like conditions, while this value was further optimized to -0.89 to recapitulate the GGF2 clinical data.

2.7. Simulations of TCZ-mediated hepatotoxicity in BIOLOGXsym

Figure S10. Simulated fraction viable hepatocytes vs. time profiles in the SimCohorts (n=4) administered TCZ alone. Simulations were performed with all mechanisms (ROS, steatosis, and explicit IL-6R effects, **A**), TCZ effects on IL-6R only (**B**), or TCZ effects on ROS and steatosis only (**C**). Each line represents each simulated individual.

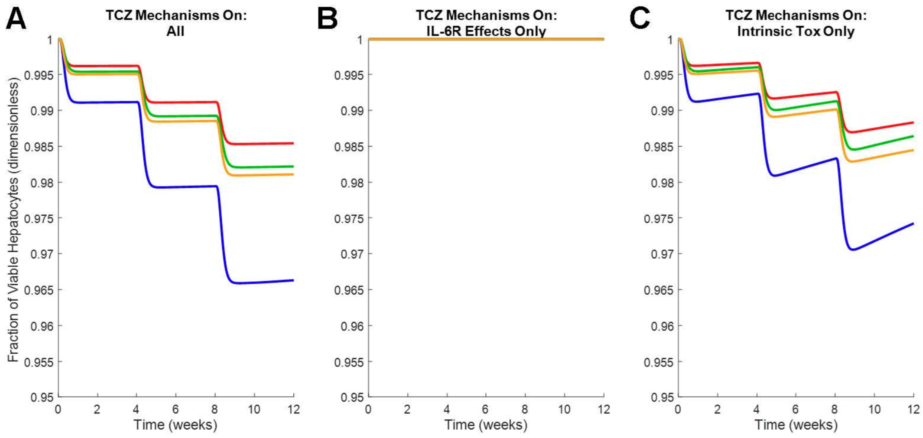

2.8. Tissue and Extracellular Matrix Remodeling

Metabolomics analysis of effluent revealed strong signatures of increased protein turnover following low dose TCZ treatment (232 µg/ml) such as free amino acids (arginine, leucine, isoleucine, valine, etc.), their post-translational derivatives (including N2-diacetyllysine, N2,N6-diacetyllysine, N6-methyllysine, N6, N6,N6-trimethyllysine, N-acetyl-histidine, dimethylarginine (SDMA + ADMA)), and dipeptides (glycylleucine, phenylalanylalanine, and valylleucine) (Figure S11). 3-methyl histidine, a marker of tissue protein breakdown[42] did not change significantly with low TCZ dose. However, higher TCZ dose showed significant decrease at day 10, indicating tissue decay in response to TCZ toxicity. Monoclonal antibodies like TCZ, are catabolized into amino acids and small peptides to facilitate their elimination from the body. Therefore, marked increases in many amino acid derivatives and dipeptides could be a cumulative result of TCZ catabolism, in addition to TCZ-induced change in tissue protein turnover.

Moreover, changes in constituents of glycosaminoglycans, a major component of extracellular matrix (ECM) were observed following TCZ treatment including N-acetylneuraminate and N-glycolylneuraminate alongside other sugars such as erythronate and glucuronate. It is noted that these changes were more pronounced with high dose TCZ (725 µg/ml). Another important signature associated with changes in ECM is increased levels of N6-carboxymethyllysine, an advanced glycation end product (AGE), with low dose TCZ treatment (Figure S11). AGEs are glycated proteins or lipids. Elevated levels of AGEs have been shown to be positively associated with the severity of NAFLD-associated steatosis[43].

Figure S11. Statistical heat map, pathway diagram, and boxplots of select metabolites associated with tissue remodeling within the spent media from the LAMPS models. Within the heatmap, trending (0.05 < p < 0.10) and significant (p ≤ 0.05) elevations are indicated by pink and red, respectively, while trending and significant reductions are represented by light blue and dark blue, respectively.

Sub Pathway	Biochemical Name	232 µg/ml TCZ D6 / Vehicle_D6	232 µg/ml TCZ D10 / Vehicle_D10	725 µg/ml TCZ D6 / Vehicle_D6	725 µg/ml TCZ D10 / Vehicle_D10
Lysine Metabolism	lysine	1.78	1.56	0.43	0.38
	N2-acetyllysine	15.91	9.42	1.00	2.67
	N6-acetyllysine	177.55	26.25	9.34	15.50
	N2,N6-diacetyllysine	142.49	158.49	1.07	19.33
Histidine Metabolism	3-methylhistidine	1.04	0.91	0.49	0.50
	N-acetylhistidine	41.48	41.96	0.99	18.11
Leucine, Isoleucine and Valine Metabolism	leucine	0.58	0.32	0.41	0.30
	N-acetylleucine	49.47	86.37	0.61	14.70
	isoleucine	0.51	0.27	0.43	0.28
	N-acetylisoleucine	6.37	10.69	0.82	5.66
	valine	0.58	0.30	0.38	0.27
	N-acetylvaline	90.21	127.75	0.73	59.08
Arginine and Proline Metabolism	arginine	0.60	0.34	0.47	0.33
	N-acetylarginine	68.56	77.19	0.87	11.63
	dimethylarginine (SDMA + ADMA)	0.63	0.28	0.55	0.28
	trans-4-hydroxyproline	1.38	0.37	0.50	3.69
	pro-hydroxy-pro	1.21	0.28	0.41	2.21
Dipeptide	alanylleucine	110.07	131.79	1.00	52.97
	glycylleucine	206.00	106.89	1.00	74.01
	leucylalanine	79.38	121.92	2.40	29.90
	phenylalanylalanine	98.84	200.49	0.88	36.51
	valylleucine	88.90	115.43	1.00	12.58
Aminosugar Metabolism	N-acetylneuraminate	0.96	0.93	0.85	0.84
	erythronate	1.32	1.33	0.45	0.49
	glucuronate	0.72	0.66	0.63	0.39
	N-glycolylneuraminate	0.98	0.94	0.85	0.83
Advanced Glycation End-product	N6-carboxymethyllysine	26.10	31.04	0.60	4.15

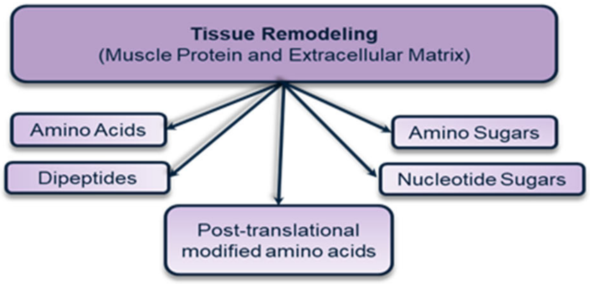

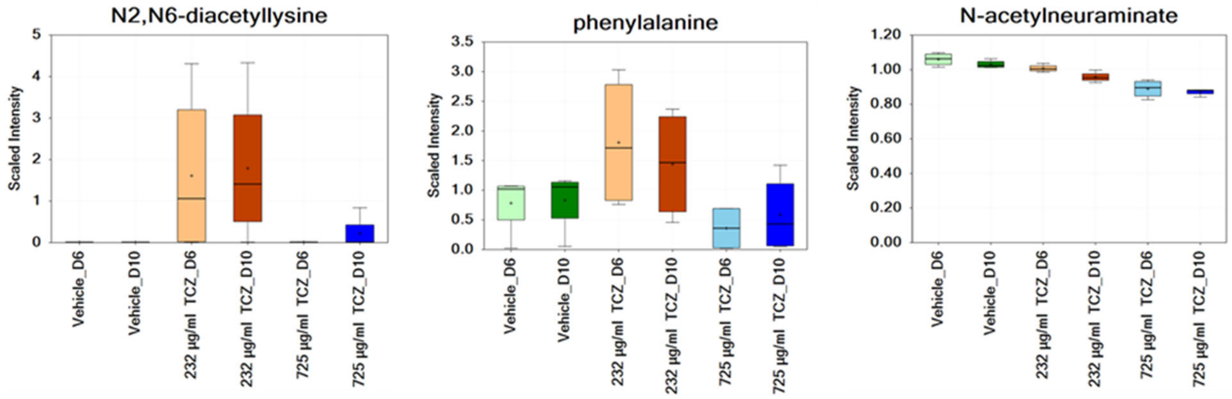

References

1. Evans, A.; Bridgewater, B.; Liu, Q.; Mitchel, A.; Robinson, R. High Resolution Mass Spectrometry Improves Data Quantity and Quality as Compared to Unit Mass Resolution Mass Spectrometry in High- Throughput Profiling Metabolomics. *Metabolomics* **2014**, *4*, 132, doi:10.4172/2153-0769.1000132.
2. Dehaven, C.D.; Evans, A.M.; Dai, H.; Lawton, K.A. Organization of GC/MS and LC/MS Metabolomics Data into Chemical Libraries. *J. Cheminformatics* **2010**, *2*, 9, doi:10.1186/1758-2946-2-9.
3. Woodhead, J.L.; Yang, K.; Siler, S.Q.; Watkins, P.B.; Brouwer, K.L.R.; Barton, H.A.; Howell, B.A. Exploring BSEP Inhibition-Mediated Toxicity with a Mechanistic Model of Drug-Induced Liver Injury. *Front. Pharmacol.* **2014**, *5*, 240, doi:10.3389/fphar.2014.00240.
4. Woodhead, J.L.; Yang, K.; Brouwer, K.L.R.; Siler, S.Q.; Stahl, S.H.; Ambroso, J.L.; Baker, D.; Watkins, P.B.; Howell, B.A. Mechanistic Modeling Reveals the Critical Knowledge Gaps in Bile Acid-Mediated DILI. *CPT Pharmacomet. Syst. Pharmacol.* **2014**, *3*, e123, doi:10.1038/psp.2014.21.
5. Balmagiya, T.; Rozovski, S.J. Thermoregulation in Young Adult Rats during Short- and Long-Term Protein Malnutrition. *J. Nutr.* **1983**, *113*, 228–238.
6. Ussher, J.R.; Koves, T.R.; Cadete, V.J.J.; Zhang, L.; Jaswal, J.S.; Swyrd, S.J.; Lopaschuk, D.G.; Proctor, S.D.; Keung, W.; Muoio, D.M.; et al. Inhibition of de Novo Ceramide Synthesis Reverses Diet-Induced Insulin Resistance and Enhances Whole-Body Oxygen Consumption. *Diabetes* **2010**, *59*, 2453–2464, doi:10.2337/db09-1293.
7. Gonzales-Pacheco, D.M.; Buss, W.C.; Koehler, K.M.; Woodside, W.F.; Alpert, S.S. Energy Restriction Reduces Metabolic Rate in Adult Male Fisher-344 Rats. *J. Nutr.* **1993**, *123*, 90–97.
8. Lazzer, S.; Bedogni, G.; Lafortuna, C.L.; Marazzi, N.; Busti, C.; Galli, R.; De Col, A.; Agosti, F.; Sartorio, A. Relationship between Basal Metabolic Rate, Gender, Age, and Body Composition in 8,780 White Obese Subjects. *Obes. Silver Spring Md* **2010**, *18*, 71–78, doi:10.1038/oby.2009.162.
9. Wang, Z.; Ying, Z.; Bosy-Westphal, A.; Zhang, J.; Heller, M.; Later, W.; Heymsfield, S.B.; Müller, M.J. Evaluation of Specific Metabolic Rates of Major Organs and Tissues: Comparison between Men and Women. *Am. J. Hum. Biol. Off. J. Hum. Biol. Counc.* **2011**, *23*, 333–338, doi:10.1002/ajhb.21137.
10. Schulz, A.R. Computer-Based Method for Calculation of the Available Energy of Proteins. *J. Nutr.* **1975**, *105*, 200–207.
11. Howell, B.A.; Yang, Y.; Kumar, R.; Woodhead, J.L.; Harrill, A.H.; Clewell, H.J., 3rd; Andersen, M.E.; Siler, S.Q.; Watkins, P.B. In Vitro to in Vivo Extrapolation and Species Response Comparisons for Drug-Induced Liver Injury (DILI) Using DILIsym™: A Mechanistic, Mathematical Model of DILI. *J. Pharmacokinet. Pharmacodyn.* **2012**, *39*, 527–541, doi:10.1007/s10928-012-9266-0.
12. Woodhead, J.L.; Howell, B.A.; Yang, Y.; Harrill, A.H.; Clewell, H.J., 3rd; Andersen, M.E.; Siler, S.Q.; Watkins, P.B. An Analysis of N-Acetylcysteine Treatment for Acetaminophen Overdose Using a Systems Model of Drug-Induced Liver Injury. *J. Pharmacol. Exp. Ther.* **2012**, *342*, 529–540, doi:10.1124/jpet.112.192930.
13. Howell, B.A.; Siler, S.Q.; Shoda, L.K.M.; Yang, Y.; Woodhead, J.L.; Watkins, P.B. A Mechanistic Model of Drug-Induced Liver Injury AIDS the Interpretation of Elevated Liver Transaminase Levels in a Phase I Clinical Trial. *CPT Pharmacomet. Syst. Pharmacol.* **2014**, *3*, e98, doi:10.1038/psp.2013.74.
14. Yang, K.; Battista, C.; Woodhead, J.L.; Stahl, S.H.; Mettetal, J.T.; Watkins, P.B.; Siler, S.Q.; Howell, B.A. Systems Pharmacology Modeling of Drug-Induced Hyperbilirubinemia: Differentiating Hepatotoxicity and Inhibition of Enzymes/Transporters. *Clin. Pharmacol. Ther.* **2017**, *101*, 501–509, doi:10.1002/cpt.619.
15. Baran, P.; Hansen, S.; Waetzig, G.H.; Akbarzadeh, M.; Lamertz, L.; Huber, H.J.; Ahmadian, M.R.; Moll, J.M.; Scheller, J. The Balance of Interleukin (IL)-6, IL-6·soluble IL-6 Receptor (SIL-6R), and IL-

6·sIL-6R·sgp130 Complexes Allows Simultaneous Classic and Trans-Signaling. *J. Biol. Chem.* **2018**, *293*, 6762–6775, doi:10.1074/jbc.RA117.001163.
16. Schmidt-Arras, D.; Rose-John, S. IL-6 Pathway in the Liver: From Physiopathology to Therapy. *J. Hepatol.* **2016**, *64*, 1403–1415, doi:10.1016/j.jhep.2016.02.004.
17. Lin, Z.-Q.; Kondo, T.; Ishida, Y.; Takayasu, T.; Mukaida, N. Essential Involvement of IL-6 in the Skin Wound-Healing Process as Evidenced by Delayed Wound Healing in IL-6-Deficient Mice. *J. Leukoc. Biol.* **2003**, *73*, 713–721, doi:10.1189/jlb.0802397.
18. Soehnlein, O.; Lindbom, L.; Weber, C. Mechanisms Underlying Neutrophil-Mediated Monocyte Recruitment. *Blood* **2009**, *114*, 4613–4623, doi:10.1182/blood-2009-06-221630.
19. Marino, M.; Scuderi, F.; Provenzano, C.; Scheller, J.; Rose-John, S.; Bartoccioni, E. IL-6 Regulates MCP-1, ICAM-1 and IL-6 Expression in Human Myoblasts. *J. Neuroimmunol.* **2008**, *196*, 41–48, doi:10.1016/j.jneuroim.2008.02.005.
20. Scheller, J.; Chalaris, A.; Schmidt-Arras, D.; Rose-John, S. The Pro- and Anti-Inflammatory Properties of the Cytokine Interleukin-6. *Biochim. Biophys. Acta* **2011**, *1813*, 878–888, doi:10.1016/j.bbamcr.2011.01.034.
21. Fazel Modares, N.; Polz, R.; Haghighi, F.; Lamertz, L.; Behnke, K.; Zhuang, Y.; Kordes, C.; Häussinger, D.; Sorg, U.R.; Pfeffer, K.; et al. IL-6 Trans-Signaling Controls Liver Regeneration After Partial Hepatectomy. *Hepatol. Baltim. Md* **2019**, *70*, 2075–2091, doi:10.1002/hep.30774.
22. Peters, M.; Blinn, G.; Jostock, T.; Schirmacher, P.; Meyer zum Büschenfelde, K.H.; Galle, P.R.; Rose-John, S. Combined Interleukin 6 and Soluble Interleukin 6 Receptor Accelerates Murine Liver Regeneration. *Gastroenterology* **2000**, *119*, 1663–1671, doi:10.1053/gast.2000.20236.
23. Jover, R.; Bort, R.; Gómez-Lechón, M.J.; Castell, J.V. Down-Regulation of Human CYP3A4 by the Inflammatory Signal Interleukin-6: Molecular Mechanism and Transcription Factors Involved. *FASEB J. Off. Publ. Fed. Am. Soc. Exp. Biol.* **2002**, *16*, 1799–1801, doi:10.1096/fj.02-0195fje.
24. Dickmann, L.J.; Patel, S.K.; Rock, D.A.; Wienkers, L.C.; Slatter, J.G. Effects of Interleukin-6 (IL-6) and an Anti-IL-6 Monoclonal Antibody on Drug-Metabolizing Enzymes in Human Hepatocyte Culture. *Drug Metab. Dispos. Biol. Fate Chem.* **2011**, *39*, 1415–1422, doi:10.1124/dmd.111.038679.
25. Yang, J.; Hao, C.; Yang, D.; Shi, D.; Song, X.; Luan, X.; Hu, G.; Yan, B. Pregnane X Receptor Is Required for Interleukin-6-Mediated down-Regulation of Cytochrome P450 3A4 in Human Hepatocytes. *Toxicol. Lett.* **2010**, *197*, 219–226, doi:10.1016/j.toxlet.2010.06.003.
26. Long, T.J.; Cosgrove, P.A.; Dunn, R.T.; Stolz, D.B.; Hamadeh, H.; Afshari, C.; McBride, H.; Griffith, L.G. Modeling Therapeutic Antibody-Small Molecule Drug-Drug Interactions Using a Three-Dimensional Perfusable Human Liver Coculture Platform. *Drug Metab. Dispos. Biol. Fate Chem.* **2016**, *44*, 1940–1948, doi:10.1124/dmd.116.071456.
27. Siewert, E.; Bort, R.; Kluge, R.; Heinrich, P.C.; Castell, J.; Jover, R. Hepatic Cytochrome P450 Down-Regulation during Aseptic Inflammation in the Mouse Is Interleukin 6 Dependent. *Hepatol. Baltim. Md* **2000**, *32*, 49–55, doi:10.1053/jhep.2000.8532.
28. Li, S.-Q.; Zhu, S.; Han, H.-M.; Lu, H.-J.; Meng, H.-Y. IL-6 Trans-Signaling Plays Important Protective Roles in Acute Liver Injury Induced by Acetaminophen in Mice. *J. Biochem. Mol. Toxicol.* **2015**, *29*, 288–297, doi:10.1002/jbt.21708.
29. Hakkola, J.; Hu, Y.; Ingelman-Sundberg, M. Mechanisms of Down-Regulation of CYP2E1 Expression by Inflammatory Cytokines in Rat Hepatoma Cells. *J. Pharmacol. Exp. Ther.* **2003**, *304*, 1048–1054, doi:10.1124/jpet.102.041582.
30. Long, T.J.; Cosgrove, P.A.; Dunn, R.T.; Stolz, D.B.; Hamadeh, H.; Afshari, C.; McBride, H.; Griffith, L.G. Modeling Therapeutic Antibody-Small Molecule Drug-Drug Interactions Using a Three-Dimensional Perfusable Human Liver Coculture Platform. *Drug Metab. Dispos. Biol. Fate Chem.* **2016**, *44*, 1940–1948, doi:10.1124/dmd.116.071456.

31. Shoda, L.K.; Battista, C.; Siler, S.Q.; Pisetsky, D.S.; Watkins, P.B.; Howell, B.A. Mechanistic Modelling of Drug-Induced Liver Injury: Investigating the Role of Innate Immune Responses. *Gene Regul. Syst. Biol.* **2017**, *11*, 1177625017696074, doi:10.1177/1177625017696074.
32. Nishimoto, N.; Kishimoto, T. Humanized Antihuman IL-6 Receptor Antibody, Tocilizumab. *Handb. Exp. Pharmacol.* **2008**, 151–160, doi:10.1007/978-3-540-73259-4_7.
33. Igawa, T.; Ishii, S.; Tachibana, T.; Maeda, A.; Higuchi, Y.; Shimaoka, S.; Moriyama, C.; Watanabe, T.; Takubo, R.; Doi, Y.; et al. Antibody Recycling by Engineered PH-Dependent Antigen Binding Improves the Duration of Antigen Neutralization. *Nat. Biotechnol.* **2010**, *28*, 1203–1207, doi:10.1038/nbt.1691.
34. Glassman, P.M.; Chen, Y.; Balthasar, J.P. Scale-up of a Physiologically-Based Pharmacokinetic Model to Predict the Disposition of Monoclonal Antibodies in Monkeys. *J. Pharmacokinet. Pharmacodyn.* **2015**, *42*, 527–540, doi:10.1007/s10928-015-9444-y.
35. Zhang, X.; Georgy, A.; Rowell, L. Pharmacokinetics and Pharmacodynamics of Tocilizumab, a Humanized Anti-Interleukin-6 Receptor Monoclonal Antibody, Following Single-Dose Administration by Subcutaneous and Intravenous Routes to Healthy Subjects. *Int. J. Clin. Pharmacol. Ther.* **2013**, *51*, 443–455, doi:10.5414/CP201819.
36. Abdallah, H.; Hsu, J.C.; Lu, P.; Fettner, S.; Zhang, X.; Douglass, W.; Bao, M.; Rowell, L.; Burmester, G.R.; Kivitz, A. Pharmacokinetic and Pharmacodynamic Analysis of Subcutaneous Tocilizumab in Patients With Rheumatoid Arthritis From 2 Randomized, Controlled Trials: SUMMACTA and BREVACTA. *J. Clin. Pharmacol.* **2017**, *57*, 459–468, doi:10.1002/jcph.826.
37. Abou-Auda, H.S.; Sakr, W. Tocilizumab: A New Anti-Rheumatic Drug. *Saudi Pharm. J. SPJ Off. Publ. Saudi Pharm. Soc.* **2010**, *18*, 257–259, doi:10.1016/j.jsps.2010.07.009.
38. Lenihan, D.J.; Anderson, S.A.; Lenneman, C.G.; Brittain, E.; Muldowney, J.A.S.; Mendes, L.; Zhao, P.Z.; Iaci, J.; Frohwein, S.; Zolty, R.; et al. A Phase I, Single Ascending Dose Study of Cimaglermin Alfa (Neuregulin 1β3) in Patients With Systolic Dysfunction and Heart Failure. *JACC Basic Transl. Sci.* **2016**, *1*, 576–586, doi:10.1016/j.jacbts.2016.09.005.
39. Parry, T.J.; Ganguly, A.; Troy, E.L.; Luis Guerrero, J.; Iaci, J.F.; Srinivas, M.; Vecchione, A.M.; Button, D.C.; Hackett, C.S.; Zolty, R.; et al. Effects of Neuregulin GGF2 (Cimaglermin Alfa) Dose and Treatment Frequency on Left Ventricular Function in Rats Following Myocardial Infarction. *Eur. J. Pharmacol.* **2017**, *796*, 76–89, doi:10.1016/j.ejphar.2016.12.024.
40. Baulida, J.; Kraus, M.H.; Alimandi, M.; Di Fiore, P.P.; Carpenter, G. All ErbB Receptors Other than the Epidermal Growth Factor Receptor Are Endocytosis Impaired. *J. Biol. Chem.* **1996**, *271*, 5251–5257, doi:10.1074/jbc.271.9.5251.
41. Mosedale, M.; Button, D.; Jackson, J.P.; Freeman, K.M.; Brouwer, K.R.; Caggiano, A.O.; Eisen, A.; Iaci, J.F.; Parry, T.J.; Stanulis, R.; et al. Transient Changes in Hepatic Physiology That Alter Bilirubin and Bile Acid Transport May Explain Elevations in Liver Chemistries Observed in Clinical Trials of GGF2 (Cimaglermin Alfa). *Toxicol. Sci. Off. J. Soc. Toxicol.* **2018**, *161*, 401–411, doi:10.1093/toxsci/kfx222.
42. Long, C.L.; Dillard, D.R.; Bodzin, J.H.; Geiger, J.W.; Blakemore, W.S. Validity of 3-Methylhistidine Excretion as an Indicator of Skeletal Muscle Protein Breakdown in Humans. *Metabolism.* **1988**, *37*, 844–849, doi:10.1016/0026-0495(88)90118-7.
43. Pereira, E.N.G. da S.; Paula, D.P.; de Araujo, B.P.; da Fonseca, M. de J.M.; Diniz, M. de F.H.S.; Daliry, A.; Griep, R.H. Advanced Glycation End Product: A Potential Biomarker for Risk Stratification of Non-Alcoholic Fatty Liver Disease in ELSA-Brasil Study. *World J. Gastroenterol.* **2021**, *27*, 4913–4928, doi:10.3748/wjg.v27.i29.4913.
